# Osteoporosis Therapy With Denosumab in Organ Transplant Recipients

**DOI:** 10.3389/fendo.2018.00162

**Published:** 2018-04-17

**Authors:** Jana Brunova, Simona Kratochvilova, Jitka Stepankova

**Affiliations:** Institute for Clinical and Experimental Medicine (IKEM), Prague, Czechia

**Keywords:** osteoporosis, denosumab, solid organ transplantation, monoclonal antibody, dual energy X-ray absorptiometry

## Abstract

**Objective:**

Osteoporosis and fragility fractures represent serious complications for the solid organ transplant population. The recommended osteoporosis therapy for organ recipients involves supplementation with calcium and vitamin D and bisphosphonate administration. However, these options can prove limited for patients with impaired renal function. An alternative therapy option is offered by denosumab, a monoclonal antibody that targets receptor activator of nuclear factor kappa-B ligand.

**Patients and methods:**

We evaluated 63 patients with osteoporosis (23 males and 40 females, age 56.4 ± 13.1 years) following solid organ transplantation (15 diabetic patients after simultaneous transplantation of the kidney and pancreas, 34 patients after kidney transplantation, and 14 patients with liver grafts). Osteoporosis was diagnosed according to standard DEXA examination using the Lunar Prodigy apparatus. Transplanted patients with impaired renal function were treated for osteoporosis of the lumbar spine (L-spine) and/or proximal femur with calcium and vitamin D supplementation and 60 mg of denosumab every 6 months between the years 2012 and 2017. The mean duration of the therapy was 1.65 ± 0.7 years.

**Results:**

After denosumab therapy, L-spine *T*-scores improved across the whole group, ranging from −2.7 ± 0.09 to −1.8 ± 1.0 (*p* < 0.001). *T*-score values for the proximal femur increased from −2.5 ± 0.8 to −2.0 ± 0.7 after the therapy (*p* < 0.01). We observed only a mild, statistically insignificant improvement in distal forearm *T*-scores. The mean increase in L-spine bone mineral density (BMD) was 11.5 ± 6.2% in subjects with osteoporosis at this site and 10.4 ± 6.1% in the case of all patients. BMD of the proximal femur increased by 10.4 ± 8.3% in patients with osteoporosis and by 7.5 ± 7.3% in all patients. Denosumab therapy decreased the prevalence of osteoporosis in the L-spine from 75 to 27% (*p* < 0.001) and proximal femur osteoporosis from 54 to 36% (*p* < 0.05). Denosumab therapy reduced elevated levels of osteocalcin and beta-crosslaps (βCTX) in comparison with baseline levels (*p* < 0.001) across the whole group of graft recipients.

**Conclusion:**

Denosumab therapy was well-tolerated and improved bone density in our group of solid organ transplant recipients. The indications are that denosumab could be a viable therapeutic option for transplanted patients with osteoporosis, especially in those with renal function impairment or bisphosphonate intolerance.

## Introduction

Osteoporosis is among the complications that have become more prevalent with the increased survival of patients after solid organ transplantation (Tx). The main consequence of osteoporosis is bone fracture, which is associated with increased morbidity and mortality rates. The pathogenesis of transplant-associated osteoporosis is multifactorial and involves altered bone metabolism during the pre-transplant period, posttransplant bone loss caused by immunosuppressive therapy and corticosteroids, persistent hyperparathyroidism (in renal graft recipients), and vitamin D deficiency (1). Currently used immunosuppressive agents have yielded favorable survival outcomes. However, the common complications of immunosuppressive therapy include the following: infections, malignancies, kidney damage, and skeletal impairment with increased risk of bone fracture (2–4). Posttransplantation bone loss is greatest in the first 6–12 months and, according to some studies, incidence of fractures can exceed 40% (5). The use of glucocorticoids, especially at high doses during the early posttransplant period, leads to a decrease in bone density, early osteoblast apoptosis, a reduction in the bone formation rate, and prolonged mineralization lag time (6, 7). Bone disease after liver transplantation can cause high incidence of skeletal fractures, ranging from 24 to 65% depending on the series (8). In the later posttransplant period, bone mineral density (BMD) can stabilize or even improve depending on allograft function (9). However, further bone loss is associated with persistent hyperparathyroidism, which mainly occurs in kidney graft recipients (10), vitamin D deficiency, rejection episodes, and persistent impairment of graft function (11, 12). The recommended osteoporosis therapy for organ recipients involves supplementation with calcium and vitamin D and administration of bisphosphonates (2, 13). Unfortunately, one limitation of bisphosphonate therapy is that it can impair renal function, which is frequent during the posttransplant period. A possible solution is treatment with denosumab, a monoclonal antibody that targets receptor activator of nuclear factor kappa-B ligand (RANKL). The aim of our study was to provide data on the efficacy of denosumab treatment in subjects after solid organ transplantation and to redress the scarcity of investigative data on its use in these subjects thus far (14, 15).

## Patients and Methods

In this retrospective study, we evaluated 63 patients (23 males and 40 females, aged 56.5 ± 13.1 years) after solid organ transplantation. The patients were treated for osteoporosis with 60 mg of denosumab every 6 months between the years 2012 and 2017. The period of time since previous transplantations ranged between 0 and 24 years (mean 6.4 ± 6.3 years) upon beginning denosumab therapy. Fifteen diabetic patients underwent simultaneous transplantation of the kidney and pancreas, 34 patients underwent kidney transplantation, and 14 patients underwent liver transplantation. All patients were treated with immunosuppressive agents, including a calcineurin antagonist, mycophenolate mofetil, sirolimus, and glucocorticoids in various doses following the transplantation. A recommended daily corticoid dose of 2.5–10 mg of prednisone was administered as a treatment for 39/63 (62%) patients at the time of introducing denosumab therapy. The average prednisone dose was 5 mg/day in the case of 26/39 treated subjects.

Osteoporosis was confirmed by standard dual energy X-ray absorptiometry (DXA) examination using the same device and software (Lunar Prodigy Primo, GE Healthcare Lunar, Madison, WI, USA) over the whole study period. Instrument quality control on the DXA scanner was performed by daily scanning of a spine phantom (QA Phantom BMD 1.002 g/cm^2^, BMC 25.07 g, area 25.01 cm^2^) as a routine procedure, with an absence of machine drift throughout the study and with a BMD coefficient variation of 0.31–0.32%. Scan time and radiation dosage were as follows: AP lumbar spine (L-spine): 60 s and 42 μGy; proximal femur: 60 s and 42 μGy. The coefficients of variation for BMD measurements were 1% for the L spine and, again, 1% for the proximal femur. The results were expressed as *T*-scores (the SD from the mean BMD for a young healthy population) in accordance with World Health Organization criteria for the definition of osteoporosis (*T* score ≤ −2.5 SD) and osteopenia (*T* score < −1 and > −2.5 SD). BMD evaluation by DXA examination was performed for all treated patients in 12-month intervals.

History of osteoporotic fractures applied to 26/63 (41.3%) graft recipients. Vertebral fractures were recorded in 3 patients, hip fractures in 6 patients, and rib fractures in 3 patients, while 5 patients had history of pelvic fracture. Peripheral fractures (ankle, foot, and forearm) applied to 12 patients, while 3 patients suffered from multiple fractures (both central and peripheral). Seventeen fractures had occurred before denosumab therapy and 12 fractures during treatment, most of which were non-vertebral (11/12). Seven patients had peripheral fractures, two had hip fractures, one had a pelvic fracture, and another a rib fracture.

The criterion for the introduction of denosumab therapy was the presence of low BMD values with a *T*-score ≤ −2.5 SD at the site of the L-spine or total proximal femur, thereby impairing renal function and preventing the administration of bisphosphonates. Patients with severe graft function impairment (CKD G5) were not considered for denosumab therapy due to the risk of severe hypocalcemia. Denosumab injections were administrated in 6-month intervals to all patients; the minimum treatment duration was 1 year, while the mean duration of therapy was 1.65 ± 0.7 years. Calcium and vitamin D supplementation comprised an essential part of all patients’ regimes both before and during therapy with denosumab. Calcium was administered in a daily dose of 500–1,000 mg and vitamin D orally in a daily dose of 800–1,000 IU, eventually in combination with 1,25(OH)_2_D_3_ (0.25–0.50 μg/day). Calcitriol was added to the regime in the case of 28/63 (44%) subjects, while 13/63 (21%) patients were treated with paricalcitol.

### Laboratory Measurements

Demographic and clinical data were obtained from charts and records at the beginning and at the end of denosumab therapy as well as during the year 2017. Blood samples were taken according to standard clinical praxis after overnight fasting. Biochemical tests were measured before denosumab therapy and at the end of treatment. In patients who were still using denosumab in 2017, tests were carried out after the final injection.

Retrospective analysis of biochemical results was performed as follows: Total serum calcium (s-Ca) (normal values 2.15–2.55 mmol/L) and creatinine (normal values 49–90 µmol/L) were analyzed spectrophotometrically using automated analyzers. Kidney function was determined by measuring serum creatinine and estimated GF as CKD-EPI values. Estimated GF was calculated according to the CKD-EPI formula (16). Intact parathyroid hormone (PTH) (normal values 1.6–6.9 pmol/L) was analyzed by electrochemiluminescence immunoassay. Osteocalcin (normal values 15–46 µg/L) and beta-crosslaps (βCTX) (normal values 0.330–0.782 µg/L) were also analyzed by electrochemiluminescence immunoassay using the Cobas e 801 analyzer (Roche Diagnostics GmbH, Mannheim). Normal values for biochemical variables were taken from those indicated by the kit manufacturers. Vitamin D concentration was applicable for 46 patients, with calcitriol concentration applicable for 33 patients. 25-OH vitamin D (normal values 23–113 nmol/L or 9.2–45.2 µg/L) and the active metabolite 1,25-OH vitamin D -1,25(OH)_2_D_3_ (1,25-OH-vitamin D; calcitriol) (normal values 47–130 pmol/L or 19.6–54.3 ng/L) were measured by the RIA method (kits from DIAsource ImmunoAssays S.A., Louvain-la-Neuve, Belgium and Immunodiagnostic Systems, Boldon, UK).

The results of serum 25-OHD_3_ were classified according to the Kidney Disease Outcomes Quality Initiative (K/DOQI) clinical practice guidelines for bone metabolism and chronic kidney disease. Therefore, vitamin D deficiency was defined as a serum level <40 nmol/L (<16 μg/L), with insufficiency between 40 and 75 nmol/L (16–30 µg/L) (13).

### Statistical Analysis

Results were analyzed using JMP statistical software. Continuous variables displaying normal distribution are expressed as mean ± SD, with others as median and range. Categorical variables are described based on absolute and relative frequencies. For before and after comparisons, the paired *t*-test or one-sample Wilcoxon signed rank test was used based on data distribution for continuous variables. For discrete variables, McNemar’s test was applied. A two-tailed *p*-value of less than 0.05 was considered statistically significant.

## Results

The laboratory data of patients at baseline and after treatment are summarized in Table 1. Graft recipients in all groups had increased levels of creatinine and PTH, neither of which differed before or after denosumab therapy. The creatinine level was lowest in patients after liver Tx but the difference was not significant. The median estimated glomerular filtration rate measured in all patients at the beginning of the therapy [0.55 mL/s/1.73 m^2^ (95% CI 0.48–0.61)] did not differ from the median CKD-EPI values measured at the end of denosumab administration [0.55 mL/s/1.73 m^2^ (95% CI 0.40–0.61)]. A significant decrease in the level of osteocalcin and beta-crosslaps (βCTX) was observed after the therapy in comparison with baseline levels (*p* < 0.001) across the whole group of graft recipients (Table 2). Statistical significance was also reached in the smaller separate groups: (βCTX) difference (*p* < 0.01) for liver and simultaneous pancreas and kidney graft recipients (*p* < 0.01) and osteocalcin (*p* < 0.01) in both groups. Patients after isolated kidney Tx for βCTX (*p* < 0.001) and for osteocalcin (*p* < 0.001). A low level of vitamin 25-OHD_3_ < 40 nmol/L (<16 μg/L) was observed in the case of 7/46 (15.2%) tested patients at the beginning of therapy, but this finding only applied to 1/44 (2.3%) by the end of the therapy. An insufficient level of 25-OHD_3_ 40–75 nmol/L (16–30 µg/L) was found in 13/46 (28.2%) patients at the beginning of denosumab therapy and in 15/44 (34%) patients upon termination of denosumab therapy.

**Table 1:** Biochemical profile before and after denosumab therapy (mean ± SD).

Graft	Before therapy			After therapy			*p*
	Creatinine (μmol/L)	s-Ca (mmol/L)	PTH (pmol/L)	Creatinine (μmol/L)	s-Ca (mmol/L)	PTH (pmol/L)
Pancreas and kidney	197.1 ± 114.8	2.42 ± 0.1	10.1 ± 4.4	197.2 ± 116.3	2.4 ± 0.1	10.8 ± 7.3	NS
*N* = 15	*N* = 15	*N* = 15	*N* = 15	*N* = 15	*N* = 15	*N* = 15	

Kidney	192.8 ± 90.3	2.4 ± 0.1	14.8 ± 8.9	208.3 ± 104.9	2.46 ± 0.1	14.2 ± 9.4	NS
*N* = 34	*N* = 34	*N* = 34	*N* = 34	*N* = 34	*N* = 34	*N* = 34	

Liver	161.3 ± 30.9	2.4 ± 0.1	8.4 ± 4.1	158.2 ± 28.4	2.39 ± 0.1	8.06 ± 5.3	NS
*N* = 14	*N* = 14	*N* = 14	*N* = 14	*N* = 14	*N* = 14	*N* = 14	

Total	177.9 ± 91.7	2.4 ± 0.1	12.2 ± 7.6	186.0 ± 101.7	2.43 ± 0.1	12.0 ± 8.4	NS
*N* = 63	*N* = 63	*N* = 63	*N* = 63	*N* = 63	*N* = 63	*N* = 63	

**Table 2:** Comparison of bone turnover markers and vitamin D levels before and after denosumab therapy (medians).

Valuables	Before	After	*p*
βCTX (μg/L)	0.74 (95% CI = 0.53–0.92)	0.22 (95% CI = 0.17–0.39)	*p* < 0.001
(*N* = 60)			

Osteocalcin (μg/L)	56.6 (95% CI = 40.8–71.4)	16.7 (95% CI = 12.1–23.0)	*p* < 0.001
(*N* = 59)			

25D3 (nmol/L)	74.4 (95% CI = 60.8–86.6)	81.0 (95% CI = 62.2–97.1)	NS
(*N* = 44)			

1,25D3 (pmol/L)	102.6 (95% CI = 80.5–124)	96.2 (95% CI = 67.4–112.6)	NS
(*N* = 33)			

### Bone Mineral Density

Osteoporosis of the L-spine (BMD *T* score ≤ −2.5) was the most frequent finding, applying to 47/63 (74.6%) subjects. Osteoporosis of the proximal femur applied to 34/63 (54%) patients and osteoporosis of the distal radius (33% radius; BMD *T* score ≤ −2.5) applied to 40/63 (63.5%) patients. Osteopenia (BMD *T* score < −1 and > −2.5) of the L-spine occurred in 13/63 (20.6%) patients, of the proximal femur (BMD *T* score < −1 and > −2.5) in 28/63 (44.4%) patients, and of the distal radius in 17/63 (27%) patients. Normal bone density of the L-spine was observed in 3/63 (4.8%) patients, of the proximal femur in only 1/63 (1.6%) patients and of the distal radius in 6/63 (9.5%) subjects. A BMD *T* score ≤ −2.5 for both the L-spine and proximal femur was the case for 19/63 (30.1%) subjects and for all three examined sites (L-spine, proximal femur, distal radius) in 17/63 (27%) subjects.

Lumbar spine osteoporosis mainly affected liver and kidney graft recipients; 14/14 (100%) and 28/34 (82.3%) patients, respectively. Osteoporosis of the proximal femur was the most prevalent in patients after simultaneous pancreas and kidney transplantation [14/15 patients (93.3%)]. The presence of CKD-MBD and osteoporosis in kidney recipients after simultaneous pancreas and kidney transplantation could not be excluded because both diseases mostly coexist after transplantation. This is also applied to BMD measurements.

The mean *T*-score value of the osteoporotic L-spine at baseline was −3.1 ± 0.6 and in all patients −2.7 ± 0.9. The proximal femur *T*-score was −3.6 ± 0.6 in patients with BMD < −2.5 and −2.5 ± 0.8 in all patients. For the distal radius, the pretreatment *T*-score was −3.6 ± 1.7 at the osteoporotic site and −2.8 ± 1.2 in all patients. The *T*-score for patients with osteoporosis in all groups exceeded a −3.0 value, while the worst *T*-score results were for the distal radius in simultaneous kidney and pancreas recipients and kidney graft recipients (*T*-scores of −4.4 ± 1.2 and −3.7 ± 1.4, respectively).

After the therapy, the L-spine *T*-score improved, reaching −1.8 ± 1.0 in all patients (*p* < 0.001). Hip *T*-score values increased to −2.0.1 ± 0.7 after the therapy (*p* < 0.01). There was only a mild, statistically insignificant improvement in the distal radius *T*-score after the therapy, reaching −2.6 ± 1.5 (*p* = 0.119) (Table 3).

**Table 3:** Changes of *T* score values following denosumab therapy (mean ± SD).

Graft	Lspine (*T*score)			Proximal femur (*T*score)			Forearm (*T*score)		
	Before	After	*p*	Before	After	*p*	Before	After	*p*
Pancreas and kidney	−1.8 ± 1.7	−0.6 ± 1.8	*p* < 0.01	−3.2 ± 0.5	−2.2 ± 0.5	*p* = 0.106	−3.1 ± 1.9	−2.9 ± 1.7	*p* = 0.630
*N* = 15									

Kidney	−2.9 ± 0.7	−2.2 ± 0.7	*p* < 0.001	−2.4 ± 1.1	−2.1 ± 0.8	*p* < 0.05	−3.0 ± 1.7	−2.8 ± 1.5	*p* = 0.180
*N* = 34									

Liver	−3.1 ± 0.5	−2.2 ± 0.6	*p* < 0.001	−2.0 ± 0.9	−1.6 ± 0.8	*p* = 0.108	−2.4 ± 1.1	−2.1 ± 1.3	*p* = 0.197
*N* = 14									

Total	−2.7 ± 0.9	−1.8 ± 1.0	*p* < 0.001	−2.5 ± 0.8	−2.0 ± 0.7	*p* < 0.01	−2.8 ± 1.2	−2.6 ± 1.5	*p* = 0.119
*N* = 63									

Lumbar spine BMD improved in all treated patients, BMD of the proximal femur in 59/63 (94%) patients, and BMD of the distal radius increased in 33/63 (52%) subjects.

The mean BMD increase was 11.5 ± 6.2% for the L-spine with a *T*-score ≤ −2.5 and 10.4 ± 6.1% in all patients. BMD of the proximal femur increased by 10.4 ± 8.3% in patients with BMD ≤ −2.5 (*T*-score at this site) and by 7.5 ± 7.3% in all patients. For the distal radius with BMD ≤ −2.5, the *T*-score was 3.96 ± 10.1% and, in all patients, BMD increased to 2.34 ± 8.6% (Table 4). The best responders to denosumab therapy were as follows: (1) patients after liver transplantation localized to the L-spine, where the presence of osteoporosis decreased from 14/14 to 4/14 cases, followed by (2) kidney graft recipients also at the L-spine site and (3) patients after simultaneous pancreas and kidney transplantation localized to the L spine and proximal femur (Table 5).

**Table 4:** Bone mineral density changes (%) in L spine, hips, and forearm in osteoporotic sites and all patients.

Graft Tx	Lspine		Proximal femur		Forearm	
	OP	All	OP	All	OP	All
Pancreas and kidney	14.0 ± 6.9	9.6 ± 6.0	7.8 ± 3.52	7.5 ± 3.3	−1.0 ± 5.3	0.6 ± 5.5
*N* = 15	*N* = 5	*N* = 15	*N* = 14	*N* = 15	*N* = 9	*N* = 14

Kidney	10.9 ± 5.6	10.1 ± 5.9	10.4 ± 8.3	8.0 ± 6.7	5.0 ± 11.6	3.0 ± 10.2
*N* = 34	*N* = 28	*N* = 34	*N* = 17	*N* = 34	*N* = 24	*N* = 34

Liver	11.4 ± 7.6	11.4 ± 7.7	16.9 ± 18.5	6.2 ± 10.8	4.9 ± 4.4	2.33 ± 5.5
*N* = 14	*N* = 14	*N* = 14	*N* = 3	*N* = 14	*N* = 7	*N* = 14

Total	11.5 ± 6.2	10.4 ± 6.1	10.4 ± 8.3	7.5 ± 7.3	3.96 ± 10.1	2.34 ± 8.6
*N* = 63	*N* = 47	*N* = 63	*N* = 34	*N* = 63	*N* = 40	*N* = 62

*OP, osteoporosis*.

**Table 5:** Prevalence of osteoporosis before and after the denosumab therapy.

Organ/Tx	L spine		Proximal femur		Forearm	
	Before	After	Before	After	Before	After
Pancreas and kidney	5/15	1/15	14/15	10/15	9/15	7/15
*N* = 15	33.3%	6.7%	93.3%	66.6%	60%	46.7%

Kidney	28/34	12/34	17/34	11/34	24/34	19/34
*N* = 34	82.3%	35.3%	50%	32.3%	70.5%	55.8%

Liver	14/14	4/14	3/14	2/14	7/14	7/14
*N* = 14	100%	28.6%	21.4%	14.3%	50%	50%

Total	47/63	17/63	34/63	23/63	40/63	33/63
*N* = 63	74.6%	26.9%	53.9%	36.5%	63.5%	52.4%

Denosumab therapy decreased the prevalence of osteoporosis (determined according to *T*-scores—WHO classification) in the L-spine from 75 to 27% (48% decrease) (*p* < 0.001), in the proximal femur from 54 to 36% (18% decrease) (*p* < 0.01), and in the distal radius from 63 to 52% (11% decrease).

## Discussion

In this retrospective study, we analyzed the results of denosumab therapy in patients after solid organ transplantation. Osteoporosis and osteopenia are common complications in cases of long-term graft transplantation survival (17, 18). The different mechanisms of action offered by existing anti-osteoporotic drugs provide alternative strategies for osteoporosis treatment. Some successfully transplanted patients exhibit impaired renal function. However, bisphosphonate therapy is not recommended in such cases, as bisphosphonates are not tolerated due to their adverse side effects. Reduced renal function occurs not only in renal grafts but can also appear in patients after liver or heart Tx. Until recently, osteoporosis therapy of patients with renal impairment was limited to the adjustment of calcium-phosphate metabolism and vitamin D levels, to the management of secondary hyperparathyroidism and to optimizing the maintenance of immunosuppressive therapy. Now, however, innovative therapeutic strategies such as denosumab therapy offer promising solutions for osteoporosis treatment in these patients (19). Clinical studies indicate that long-term treatment with denosumab causes a continuous increase in BMD with low incidence of adverse effects. Denosumab has been successfully used for several years in postmenopausal osteoporosis therapy with proven lowering of bone reduction, increased BMD, and decreased vertebral and non-vertebral fracture risk (20). In contrast to other drugs that reduce bone resorption such as third-generation bisphosphonates, denosumab is a monoclonal antibody that targets RANK ligand. RANKL, which is mainly produced by osteocytes, supports osteoclast formation and activity. When denosumab binds to RANKL, it blocks its activity, decreasing the formation, function, and survival of osteoclasts and, therefore, bone resorption. These actions result in an increase in BMD and an improvement in bone strength (21). Denosumab may also have a favorable effect on posttransplant bone loss due to the effect of calcineurin inhibitors on osteoclast activity and glucocorticoid activation of the RANKL system (2). A significant advantage of denosumab therapy is that it can be used in patients with impaired renal function, and it is not known to exhibit any adverse interaction with other medications currently in use. Moreover, subcutaneous administration of denosumab every 6 months is a less burdensome option for post-Tx patients who are administered large daily doses of various medications.

Despite reports that denosumab successfully improves BMD in patients with impaired renal function (CKD G 2–4) (22), currently, only a few reports concerning the use of denosumab in osteoporosis treatment of solid organ recipients exist (23–25). In the POSTOP study, denosumab was administered to newly transplanted kidney patients to prevent bone loss, even in subjects without osteoporosis, in the early posttransplant period (14). In our patients, the time elapsed since their previous transplantations was between 0 and 24 years (mean 6.4 years), with almost 1/3 (l9/63) patients surviving for more than 10 years after Tx survival. All of them were known to have osteoporosis (defined as a persistent low BMD with a *T* score ≤ −2.5 and/or a significant decrease of BMD compared to osteoporotic values) and 41% had undergone at least one recent osteoporotic fracture. Impaired renal function in these patients did not permit bisphosphonate therapy, while standard calcium/vitamin D combination therapy was not effective enough to improve bone density. Although we were aware of the heterogeneity concerning the pre-transplant history in our transplanted patients—duration of renal failure and dialysis, attempts to treat bone loss prior to transplantation, years elapsed since Tx, number of rejection episodes, menopausal status in women, and the variety of immunosuppressive medications used, including glucocorticoid dosage—we considered the introduction of denosumab therapy the only solution to prevent further bone loss. The therapy was well-tolerated in the first patients treated and, moreover, they showed significant improvement in BMD. The number of treated patients gradually increased, with 60% of them currently continuing with denosumab treatment. The mean increase in BMD for the whole group was more than 10% for the L-spine and 7–10% for the proximal femur during the relatively short period of treatment.

The risk factors associated with antiresorptive therapy using denosumab include hypocalcemia, osteonecrosis of the jaw, and increased incidence of infection (2). Hypocalcemia mainly jeopardizes patients with more advanced renal impairment (CKD G 4–5) (26). In our study, all patients were placed on a course of calcium and/or vitamin D substitution therapy. A low level of 25OHD_3_ was found only in one patient on ongoing denosumab therapy. Mild hypocalcemia occurred in one patient with a progressive decline in renal function, but was successfully treated with a temporary increase in the usual daily calcium dose. No other person displayed clinical or laboratory signs of hypocalcemia during denosumab administration. Creatinine levels were stable up to 200 µmol/L in most of our transplanted patients throughout the course of therapy. Hypocalcemia occurs mainly during the first weeks after denosumab injection and, therefore, calcium levels should be measured thereafter. We usually monitor calcium levels within 1 month after the injection, especially in patients newly administered denosumab therapy and in those with unstable or higher creatinine values (CKD G 4), in order to negate the eventual decrease in calcium levels.

Osteonecrosis of the jaw was not observed among our denosumab-treated graft recipients. Denosumab-associated incidence of jaw osteonecrosis in patients with osteoporosis is generally very low (0.15–0.001% of persons per year of therapy). The vast majority (>90%) of cases occur in oncology patients receiving high doses of intravenous bisphosphonates or subcutaneous denosumab (27). Additional risk factors include periodontal disease, oral surgical procedures, radiation and chemotherapy, diabetes, glucocorticoid use, and smoking. The non-occurrence of jaw osteonecrosis in our monitored patients can be explained by the fact that all transplantation candidates underwent a dental sanitation programme, followed by regular dental check-ups. None of our patients suffered subtrochanteric fractures during denosumab treatment.

A recently published study (28) reported more frequent episodes of urinary infection (cystitis) in denosumab-treated *de novo* kidney transplant recipients (34.9%) versus 25.2% in non-treated patients; no difference was found among other infections. We did not observe any increased incidence of infections in denosumab-treated patients in comparison with other graft recipients monitored at our institute. Neither did we consider it apt to perform a comparative study because of the heterogeneity of our graft recipient population. In most of our patients, a significant period of time had elapsed since their last Tx (there were only three newly transplanted patients) and, consequently, all had a history of various previous infectious complications since then. We did not observe any episode of urinary infection in the three patients who were put on denosumab therapy during the first year after Tx, even though it must be stated that this number is too small to be statistically significant.

Approximately 60% of patients are still undergoing denosumab therapy. In the case of 11 patients, treatment was terminated due to a substantial increase in BMD compared to non-osteoporotic values, while in three patients, an improvement in renal function enabled the use of bisphosphonates. The BMD development in this latter group of patients will be the subject of further investigation. Denosumab therapy was discontinued in the case of three patients who were due to resume dialysis, while another set of three patients were later treated without denosumab in another hospital. Of the 63 patients in our group, 5 died while still on therapy: 3 from neuro/cardiovascular causes and 2 polymorbid patients from sepsis. Initially, we were not sure whether to prolong denosumab therapy in transplanted patients beyond a period of 2 years or in cases where there had been an improvement in the *T*-score above −2.0, as the data on application of the therapy in these instances are threadbare. On the other hand, the discontinuation of denosumab frequently leads to BMD loss and vertebral fractures. The deaths caused by sepsis in our group did not represent a difference with the usual number of fatal complications reported for those that are immune-compromised and (frequently) for polymorbid patients.

Bone density improved mainly in the L-spine across all graft recipient groups, with the highest increase at osteoporotic sites. BMD also significantly increased in the proximal femur locality. From a relative perspective, the weakest effect of denosumab was recorded in the distal forearm, in which case, BMD increased in 61% of patients and decreased or remained unchanged in 39%. However, osteoporosis at this site is generally difficult to modify using accessible antiresorptive therapy. Furthermore, parathyroid hormone (PTH) values, which remained increased in all patients, preferentially exert a catabolic effect on the cortical bone. PTH decreases by 50% about 6 months after kidney Tx but remains high in nearly 45% of graft recipients 2 years after transplantation (29). The main causes of persistent PTH elevation in transplanted patients are low vitamin D levels, hyperplasia, or adenoma of the parathyroid gland (which mainly affects kidney graft recipients) and impaired renal function. The mildly increased PTH levels observed in our study probably reflected our patients’ impaired renal function, with no significant difference observed after denosumab therapy. The modest increase in PTH may represent an appropriate adaptive response to declining renal function due to phosphaturic effects and increased bone resistance to PTH (30). We did not observe the parathyroid adenomas associated with more severe hyperparathyroidism. Parathyroid adenomas, which are less common in kidney graft recipients, have recently been successfully prevented with paricalcitol therapy introduced during the pretransplant period. We observed no significant changes in parathormone or creatinine levels as a result of denosumab therapy. On the other hand, the decrease of accelerated bone turnover markers (BTM) was significant, a finding that correlated with improvement in bone density after therapy. Monitoring these markers is restricted by the fact that there are currently no reference standards for transplanted patients. Additionally, BTM are impacted by a number of factors such as age, gender, medication, and renal impairment (2). The elevation of mean BTM in our patients before denosumab therapy along with the relatively non-suppressed osteocalcin and βCTX levels below expected subnormal values were influenced by impaired renal clearance and, therefore, could not completely reflect bone turnover. However, comparing these values can be a valuable additional method in the early assessment of treatment responses.

## Conclusion

Denosumab therapy markedly improved BMD in solid organ recipients. A significant increase in *T*-scores was observed in the L-spine and hips. The prevalence of osteoporosis (diagnosed according to *T*-score values) in the L-spine decreased by 48% and in the hips by 18%, even following the relatively short duration of therapy. Denosumab therapy was well-tolerated, and we did not observe any serious complications such as osteonecrosis of the jaw or subtrochanteric fractures, or indeed, any particular increase in infectious complications. These findings indicate that denosumab could be a possible useful option for osteoporosis therapy in the organ transplant population, especially in those with renal impairment. Nevertheless, further designated studies must be conducted to demonstrate its long-term safety and the probable beneficial effects on fracture risk in transplanted patients with osteoporosis.

## Ethics Statement

This study was retrospective, and we analyzed results of patients who were already treated in past or currently for osteoporosis with denosumab at our Endocrine and Nephrology out-patient clinics. The therapy with Denosumab was introduced in subjects with renal impairment and with osteoporosis of L spine and/or hips as an alternative treatment to bisphosphonates. More than 40% of those patients had a history of fragility fracture. All patients were regularly physically examined and their laboratory results were controlled. All patients treated in our hospital gave routinely informed consent with examination and treatment. The data for our manuscript were retrospectively collected from patients’ files. However, biochemical and DEXA results were strictly anonymized during the evaluation process. The data are presented as means and medians eventually in percentage and, therefore, the individual patients are not absolutely traceable.

## Author Contributions

JB: the main author of manuscript; she was involved in the patient’ therapy with denosumab and their follow up at out-patient clinic, and evaluated the DEXA examinations and she collected all results from patients’ charts; she is responsible for the discussion of results and conclusions, which were made. SK regularly followed the osteoporotic patients treated with denosumab at out-patient basis, and was also involved in evaluation of repeated DEXA examination of all subjects; she contributed to the discussion of the results. JS regularly examined and treated patients after the kidney transplantation with denosumab at nephrology clinic.

## Conflict of Interest Statement

The authors declare that the research was conducted in the absence of any commercial or financial relationships that could be construed as a potential conflict of interest. The reviewer JS and VZ and handling Editor declared their shared affiliation.

## References

[1] Ebeling PR Approach to the patient with transplantation-related bone loss. J Clin Endocrinol Metab (2006) 94:1483–90. 10.1210/jc.2009-0205 19420272

[2] Early C Stuckey L Tischer S Osteoporosis in the adult solid organ transplant population: underlying mechanism and available treatment options. Osteoporos Int (2016) 27:1425–40. 10.1007/s00198-015-3367-8 26475288

[3] Gregorini M Sileno G Pattonieri EF Corradetti V Abeli M Ticozzelli E Understanding bone damage after kidney transplantation: a retrospective monocentric cross sectional analysis. Transplant Proc (2017) 49:650–7. 10.1016/j.transproceed.2017.02.023 28457365

[4] Alis G Alis M Erturk T Karayagiz AH Berber I Cakir U. Evaluation of bone disease in kidney transplant recipients. Transplant Proc (2017) 49:509–11. 10.1016/j.transproceed.2017.01.040 28340823

[5] Alshayeb HM Josephson MA Sprague SM. CKD-mineral and bone disorder management in kidney transplant recipients. Am J Kidney Dis (2013) 61:310–25. 10.1053/j.ajkd.2012.07.022 23102732

[6] Löfdahl E Radegran G Osteoporosis following heart transplantation and immunosuppressive therapy. Transplant Rev (2017) 31:232–9. 10.1016/j.trre.2017.08.002 28865930

[7] Damasiewitz MJ Ebeling PR Management of mineral and bone disorders in renal transplant recipient. Nephrology (2017) 22(Suppl 2):65–9. 10.1111/nep.13028 28429555

[8] Monegal A Navasa M Peris P Colmenero J Cuervo A Muxí A. Bone disease in patients awaiting liver transplantation. Has the situation improved in the last two decades? Calcif Tissue Int (2013) 93(6):571–6. 10.1007/s00223-013-9797-4 24065305

[9] Carlini RG Rojas E Weisinger JR Lopez M Martinis R Arminio A Bone disease in patients with long-term renal transplantation and normal renal function. Am J Kidney Dis (2000) 36:160–6. 10.1053/ajkd.2000.8289 10873886

[10] Perrin P Caillard S Javier RM Braun L Heibel F Borni-Duval C Persistent hyperparathyroidism is a major risk factor for fractures in the 5 years after kidney transplantation. Am J Transplant (2013) 13:2653–63. 10.1111/ajt.12425 24034142

[11] Kantalar-Zadeh K Molnar MZ Kovesdy CP Mucsi I Bunnapradist S Management of mineral and bone disorders after kidney transplantation. Curr Opin Nephrol Hypertens (2012) 21(4):389–403. 10.1097/MNH.0b013e3283546ee0 22614626 PMC3532932

[12] Kulak CA Borba VZ Kulak J Jr Custodio MR. Osteoporosis after transplantation. Curr Osteoporos Rep (2012) 10:48–55. 10.1007/s11914-011-0083-y 22167576

[13] National Kidney Foundation. K/DOQI clinical practice guidelines for bone metabolism and disease in chronic kidney disease. Am J Kidney Dis (2003) 42:S1–201. 10.1016/S0272-6386(03)00905-3 14520607

[14] Bonani M Frey D Brockmann J Fehr T Mueller TF Saleh L Effect of twice-yearly denosumab on prevention of bone mineral density loss in de novo kidney transplant recipients: a randomized controlled trial. Am J Transplant (2016) 16:1882–91. 10.1111/ajt.13692 26713403

[15] Bonani M Meyer U Frey D Graf N Bischoff-Ferrari HA Wüthrich RP. Effect of denosumab on peripheral compartmental bone density, microarchitecture and estimated bone strength in de novo kidney transplant recipients. Kidney Blood Press Res (2016) 4:614–22. 10.1159/000447930 27622692

[16] Levey AS Stevens LA Schmid CH Zhang YL Castro AF III Feldman HI A new equation to estimate glomerular filtration rate. Ann Intern Med (2009) 150:604–12. 10.7326/0003-4819-150-9-200905050-00006 19414839 PMC2763564

[17] Rocha A Martins LS Malheiro J Dores J Santos C Henriques C. Changes in bone mineral density following long-term simultaneous pancreas-kidney transplantation. J Bone Miner Metab (2016) 34(2):209–15. 10.1007/s00774-015-0657-3 25837429

[18] Dounousi E Leivaditis K Eleftheriadis T Liakopoulos V. Osteoporosis after renal transplantation. Int Urol Nephrol (2015) 47:503–11. 10.1007/s11255-014-0862-3 25384432

[19] Faienza MF Chiarito M D’amato G Colaiani G Colucci S Grano M Monoclonal antibodies for treating osteoporosis. Expert Opin Biol Ther (2017) 7:1–9. 10.1080/14712598.2018.1401607 29113523

[20] Papapoulos S Lippuner K Roux C Lin CJ Kendler DL Lewiecki EM The effect of 8 or 5 years of denosumab treatment in postmenopausal women with osteoporosis: results from the FREEDOM extension study. Osteoporos Int (2015) 26:2773–83. 10.1007/s00198-015-3234-7 26202488 PMC4656716

[21] Cummings SR San Martin J McClung MR Siris ES Eastell R Reid IR Denosumab for prevention of fractures in postmenopausal women with osteoporosis. New Engl J Med (2009) 361(8):756–65. 10.1056/NEJMoa0809493 19671655

[22] Jamal SA West SL Miller PD. Bone and kidney disease: diagnostic and therapeutic implications. Curr Rheumatol Rep (2012) 14:217–23. 10.1007/s11926-012-0243-9 22350608

[23] Bonani M Seaa AL Fehr T Brockmann J Schiesser M A randomized open-label clinical trial examining the effect of denosumab on the prevention of first-year bone mineral density loss after renal transplantation. Transplantation (2012) 94(10S):887 10.1097/00007890-201211271-01745

[24] Brunova J Kratochvilova S Stepankova J Denosumab in the treatment of osteoporosis in patients after organ transplantation. Osteol Bull (2016) 21(4):111–8.

[25] Wada Y Iyoda M Iseri K Arai-Nunota N Combination therapy of denosumab for renal transplant recipient with severe bone loss due to therapy-resistant hyperparathyroidism. Tohoku J Exp Med (2016) 238:205–12. 10.1620/tjem.238.205 26947314

[26] Dave V Chiang CY Booth J Mount PF. Hypocalcemia post denosumab in patients with chronic kidney disease stage 4-5. Am J Nephrol (2015) 41:129–37. 10.1159/000380960 25790847

[27] Khan A Morrison A Cheung A Hashem W Compston J. Osteonecrosis of the jaw (ONJ): diagnosis and management in 2015. Osteoporos Int (2016) 27:853–9. 10.1007/s00198-015-3335-3 26493811

[28] Bonani M Frey D de Rougemont O Mueller NJ Mueller TF Graf N Infections in de novo kidney transplant recipients treated with the RANKL inhibitor denosumab. Transplantation (2017) 101:2139–45. 10.1097/TP.0000000000001547 27798510

[29] Lou I Foley D Odorico SK Leverson G Schneider DF Sippel R How well does renal transplantation cure hyperparathyroidism? Ann Surg (2015) 262:653–9. 10.1097/SLA.0000000000001431 26366545 PMC4576689

[30] Kettler M Block GA Evenoel P Fukagawa M Herzog CA Executive summary of the 2017 KDIGO chronc kidney disease-mineral and bone disorder (CKD-MBD) guideline update: what’s changed and why it matters. Kidney Int (2017) 92:26–36. 10.1016/j.kint.2017.04.006 28646995

